# Maximizing utility of neuropsychological measures in sex‐specific predictive models of incident Alzheimer's disease in the Framingham Heart Study

**DOI:** 10.1002/alz.13500

**Published:** 2023-10-26

**Authors:** Maria Teresa Ferretti, Huitong Ding, Rhoda Au, Chunyu Liu, Sherral Devine, Sanford Auerbach, Jesse Mez, Ashita Gurnani, Yulin Liu, Antonella Santuccione, Ting Fang Alvin Ang

**Affiliations:** ^1^ Institute for Regenerative Medicine (IREM) University of Zurich Zurich Switzerland; ^2^ Women's Brain Project Guntershausen Switzerland; ^3^ Department of Anatomy and Neurobiology Boston University Chobanian & Avedisian School of Medicine Boston Massachusetts USA; ^4^ The Framingham Heart Study Boston University Chobanian & Avedisian School of Medicine Boston Massachusetts USA; ^5^ Department of Epidemiology Boston University School of Public Health Boston Massachusetts USA; ^6^ Slone Epidemiology Center Boston University Chobanian & Avedisian School of Medicine Boston Massachusetts USA; ^7^ Department of Neurology Boston University Chobanian & Avedisian School of Medicine Boston Massachusetts USA; ^8^ Department of Biostatistics Boston University School of Public Health Boston Massachusetts USA

**Keywords:** Alzheimer's disease, machine learning, neuropsychological measures, process making, sex differences

## Abstract

**INTRODUCTION:**

Sex differences in neuropsychological (NP) test performance might have important implications for the diagnosis of Alzheimer's disease (AD). This study investigates sex differences in neuropsychological performance among individuals without dementia at baseline.

**METHODS:**

Neuropsychological assessment data, both standard test scores and process coded responses, from Framingham Heart Study participants were analyzed for sex differences using regression model and Cox proportional hazards model. Optimal NP profiles were identified by machine learning methods for men and women.

**RESULTS:**

Sex differences were observed in both summary scores and composite process scores of NP tests in terms of adjusted means and their associations with AD incidence. The optimal NP profiles for men and women have 10 and 8 measures, respectively, and achieve 0.76 mean area under the curve for AD prediction.

**DISCUSSION:**

These results suggest that NP tests can be leveraged for developing more sensitive, sex‐specific indices for the diagnosis of AD.

## 1 BACKGROUND

Research has consistently shown that women are at higher lifetime risk for developing Alzheimer's disease (AD) than men.^1^, ^2^, ^3^, ^4^, ^5^ Differences in cognitive function between sexes are evident throughout adulthood and physiological aging as well.^6^ In a healthy population, women tend to outperform men in most verbal memory tasks, while men present an advantage in visuospatial tasks and navigation;^7^ these differences were observed in the elderly population too.^8^ To account for sex effects, normative data were generated for neuropsychological (NP) tests.^9^, ^10^, ^11^

Emerging research indicates that AD is a neurodegenerative disorder characterized by a progressive continuum of symptoms and stages of clinical manifestation, associated with the silent accumulation of pathological AD biomarkers.^12^ Subjective cognitive impairment (SCI) and mild cognitive impairment (MCI) start several years before AD diagnosis,^13^, ^14^, ^15^, ^16^ collectively referred as preclinical AD for this study. The ability to identify individuals with seemingly normal test scores who progress to AD is crucial for preventative strategies, early initiation of clinical interventions, and patient selection for clinical trials. There is therefore an urgent and unmet need for sensitive NP screening tools.^17^, ^18^, ^19^

Sex differences in NP tests have not been thoroughly harnessed despite numerous studies reporting them. In a study of MCI, given equal levels of hippocampal atrophy, women present with relatively preserved verbal memory compared to men.^20^ Mini‐Mental State Examination (MMSE) scores at first AD diagnosis were lower for women than men in the French National Alzheimer database, suggesting a late diagnosis in women.^21^ Overall, cognition in female AD patients deteriorated faster than male patients,^22^ suggesting a greater pathological burden at detection, leading to a faster disease progression thereafter. Indeed, adjusting NP cutoffs for sex differences would significantly increase the number of women diagnosed with MCI.^23^

NP test scores used for the detection of cognitive impairment across the AD cognitive spectrum heavily rely on measures of episodic memory and verbal functions. Because women outperform men on these tests,^24^, ^25^ subtle impairments may go undetected because of ceiling effects in screening instruments or lower threshold cut‐offs scores for impairment.

Another theory postulates women use their higher verbal skills to “mask” incipient cognitive impairments. Individuals sometimes provide process responses before coming up with the correct responses during NP assessments. An example of a process response is when participants make an initial error but subsequently correct it during the memory recall. These additional intervening responses may reflect subtle underlying cognitive changes that render the immediate provision of a correct response harder. The Boston Process Approach (BPA) tracks responses beyond the correct answers, recording behaviors such as circumlocutions, perseverations, intrusion, and so on. These responses complement standard test scores and provide a more in‐depth cognition profile of individuals via NP assessments.^26^, ^27^ Since 2005, the Framingham Heart Study (FHS) has recorded these process responses.^28^, ^29^

To the best of our knowledge, sex differences in the predictive value of NP performance, both the correct and process responses, for incident AD dementia have never been studied on a community‐based population. The FHS provides the ideal study population with BPA measures and sufficient incident AD cases after stratification by sex. This study is designed to test two main hypotheses: (1) Sex differences in NP test performance, correct and process responses, can predict AD incidence. (2) Optimal sets of metrics, with respect to NP performance, for AD incidence prediction will differ between sexes.

**RESEARCH IN CONTEXT**

1. **Systematic review**: We reviewed the literature using databases such as PubMed. Sex differences in neuropsychological (NP) test performance might have important implications for the diagnosis of cognitive disorders such as Alzheimer's disease (AD). A detailed study of sex differences in the predictive value of NP performance, considering both the correct and processes responses, for incident AD dementia has not been performed on a community‐based study population.
2. **Interpretation**: Our study investigated sex differences in NP performance, including standard tests scores and process coded responses, among individuals without dementia from the Framingham Heart Study. The results suggest that NP tests can be leveraged for developing more sensitive, sex‐specific indices for the diagnosis of preclinical AD.
3. **Future directions**: Future studies should include attempts to (1) identify the biological underpinning of such sex‐related differences in performance and strategy; and (2) extend the use of composite process scores in AD research.

## 2 METHODS

### 2.1 Definition of sex

In the FHS data, sex is self‐reported; gender was not recorded.^30^ Thus, we refer to differences between men and women as “sex differences” in the context of this paper given the methods of data collection.

### 2.2 Study participants

**FIGURE 1:**
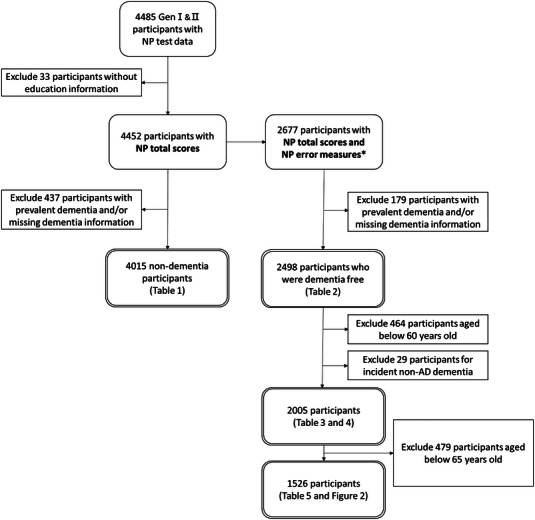
Sample selection process. *BPA started in year 2005 and 1775 were excluded due to the absence of NP error measures. AD, Alzheimer's disease; BPA, Boston Process Approach; NP, neuropsychological

FHS was initiated in 1948 and recruited an initial cohort, often referred to as the Gen 1 (Original) cohort. Subsequently, in 1971, the Gen 2 (Offspring) cohort was enrolled. The FHS features a 99% retention rate of participants, regularly returning to follow‐ups.^31^ This paper included Gen 1 and 2 participants who underwent FHS NP assessment (*n* = 4485). Among them, 33 participants were excluded due to missing education information. Participants with prevalent dementia and/or missing dementia information were also excluded—possible MCI at baseline cognitive assessment is not an exclusion criterion. Analysis was performed on the participants with NP total scores of their first FHS NP assessments (*n* = 4015) and participants with both NP total scores and NP error measures collected, available after year 2005 (*n* = 2498). Refer to Figure 1 for the sample selection flowchart.

### 2.3 Diagnostic criteria

Surveillance for incident dementia/AD was initiated in 1975, when a 20‐minute NP assessment of the Gen 1 cohort was introduced to establish a dementia‐free cohort. In 1981, cognitive screening using the MMSE was integrated into their biennial health examinations. Baseline cognitive status was determined by self‐report in 1979, with formal cognitive screening with the MMSE of the Gen 2 cohort beginning in 1991. FHS participants were also invited for more comprehensive NP testing approximately every 4 to 5 years. The BPA, which scores both correct and extraneous and process responses to NP test questions^27^ was implemented in 2005.

Every diagnosed case of dementia/AD is adjudicated through a panel, which includes at least one neurologist and one neuropsychologist, using, where available, information gathered from neurologic and NP assessments, FHS health exams, medical records, and family interviews.^32^ Details of the surveillance and diagnosis of dementia within FHS have been published previously.^33^, ^34^, ^35^ FHS diagnostic criteria of dementia are based on the Diagnostic and Statistical Manual of Mental Disorders, fourth edition (DSM‐IV), while that of AD are derived from the National Institute of Neurological and Communicative Disorders and Stroke and the Alzheimer's Disease and Related Disorders Association (NINCDS–ADRDA).^36^

### 2.4 NP tests and process measures

The FHS NP battery includes the following tests: Wechsler Memory Scale‐First Edition (WMS)–Logical Memory (LM)–Passage A, WMS Visual Reproduction (VR), WMS Paired Associate Learning (PAL),^37^ Wechsler Adult Intelligence Scale First Edition (WAIS), Digit Spans, WAIS Similarities,^38^ Boston Naming Test (BNT),^39^ Trail‐Making Test Parts A and B (Trails A and B), Finger Tapping, Verbal Fluency,^9^ Hooper Visual Organization Test,^40^ and Wide Range Achievement Test–Third Edition (WRAT‐3)–Reading.^41^

From 2005, in addition to scoring for correct responses, data of incorrect or extraneous responses (e.g., process measures), such as confabulations, perseveration, intrusions, and so on, were collected as well. The description of process measures used in this study is presented in Table S1 in supporting information. These process measures are categorized to three cognitive domains including self‐monitoring, abstract thinking, and motor. Only data from non‐demented participants was used in this analysis, as process measures of demented participants have high rates of missing data, as they were often unable to perform and/or complete tasks.

### 2.5 Statistical analyses

The Student *t* test was used to compare baseline age and the chi‐square test of independence was used to compare baseline education level—dichotomized as high school graduate and below versus beyond high school graduate, between men and women. The means for NP total scores stratified by sex, after adjusting for age and education, were generated. Trails A and B were log‐transformed due to their right‐skewed distributions. For all NP tests, except Trails A and B, higher adjusted scores reflected better cognitive performance.

Three composite process scores for cognitive functions including self‐monitoring, abstract thinking, and motor were generated by confirmatory factor analysis (CFA) based on all process measures for each cognitive domain using Mplus (version 8.3).^42^ For the binary process measures, the missing values were given the value of zero. We performed multiple imputation using the chained equations approach to impute missing values for non‐binary process measures.^43^ We developed ordered categorical transformations of the raw non‐binary process data to facilitate the development of composite scores that did not make strong assumptions about the distributions of process measures.^44^ For ordinal variables, we mapped the raw process measures onto an ascending scale of 10—the maximum value for Mplus software. A single factor model was built for each cognitive function with the WLSMV estimator. The loadings of each process measure were used as their weights to compute composite process scores.^44^

The association of individual NP total scores and NP composite process scores with incident AD was analyzed by Cox proportional hazards model for non‐demented participants who were at least 60 years of age at the time of the NP testing (*n* = 2005), stratified by sex, to avoid immortal time bias. For participants with incident AD, follow‐up time accrued from the baseline NP examination until the earliest documented date of dementia. For participants not experiencing the outcome by 2019, follow‐up time was censored at the end of 2019, the last known follow‐up date or the date of death, whichever is earlier. Both individual NP total scores and NP composite process scores were used as the main predictors in the model, with age and education as the covariates. For easier result interpretation, the composite process scores were transformed into *z* scores, with a mean of 0 and a standard deviation of 1, before being included in the model. To minimize the rate of false positives due to multiple testing, the critical alpha level was adjusted using the conventional Bonferroni approach for all analyses (refer to individual table footnotes).

For the second hypothesis, a two‐step machine learning framework was used to identify the optimal NP profiles for incident AD predictive modeling for each sex. The Minimum Redundancy Maximum Relevance (MRMR) method^45^ was used to remove the redundant or irrelevant features and generate a group of candidate feature subsets, followed by the training, using the Xgboost model,^46^ on each candidate feature subset. To minimize class imbalance and optimize the model performance, we restricted this analysis to participants > 65 years old (*n* = 1526) and implemented the Synthetic Minority Over‐Sampling Technique (SMOTE) in this analysis.^47^ The candidate subset that has the best mean area under the curve (AUC), with 10‐folder cross‐validation, for the Xgboost model was selected. The information gain in Xgboost model^48^ was selected as the measure to interpret the relative importance of each feature in the identified optimal NP profile.

Statistical analyses were performed using Python (version 3.9.7) and R software (version 4.1.2). All FHS participants provided informed consent and the study protocol was approved by the institutional review board of the Boston University Medical Center. This study follows the Strengthening the Reporting of Observational Studies in Epidemiology (STROBE) reporting guideline.

## 3 RESULTS

### 3.1 Baseline demographics

The baseline age, education level, and race/ethnicity are summarized in Table 1. On average, women were older than men (67.5 vs. 65.9, *P* < 0.0001) and fewer received beyond high school graduate education (53.6% vs. 59.5%, *P* < 0.001). Other non‐demographic characteristics can be found in Table S2 in supporting information.[Table alz13500-tbl-0002], [Table alz13500-tbl-0003]

**TABLE 1:** Demographic, and adjusted means of neuropsychological test total scores at baseline of participants without dementia.

	Gen 1 & 2 participants without dementia (*n* = 4015)			
Variables	Men (*n* = 1787)	Women (*n* = 2228)	Effect size	*P* values^+^
Age, years, mean ± SD (range)	65.9 ± 11.0 (37, 96)	67.5 ± 12.3 (34, 101)	–	<0.0001
Education, *n* (%)				<0.001
High school graduate and below	723 (40.5)	1034 (46.4)	–	
Beyond high school graduate	1064(59.5)	1194 (53.6)	–	
Race/ethnicity^**^, *n* (%)			–	0.968
Non‐Hispanic White	1765 (98.8)	2202 (98.8)		
Others	22 (1.2)	26 (1.2)		
**NP tests with female advantage**				
Logical Memory				
Immediate Recall	10.01 (0.08)	10.96 (0.07)	–0.95	< 1.0E‐10^*^
Delayed Recall	8.96 (0.09)	9.89 (0.08)	–0.93	< 1.0E‐10^*^
Recognition	9.19 (0.03)	9.48 (0.03)	–0.29	< 1.0E‐10^*^
Paired associate learning				
Immediate Recall	12.43 (0.08)	13.87 (0.07)	–1.43	< 1.0E‐10^*^
Delayed Recall	7.79 (0.04)	8.38(0.03)	–0.59	< 1.0E‐10^*^
Recognition	9.63 (0.06)	9.71 (0.05)	–0.07	0.361
Verbal fluency test				
Phonemic (F‐A‐S)	31.29 (0.52)	33.29 (0.44)	‐2.00	0.003
Category (Animals)	18.00 (0.41)	17.55 (0.37)	0.45	0.407
Trails–Log‐transformed				
Trail A	0.46 (0.00)	0.44 (0.00)	0.02	0.001^*^
Trail B	0.91 (0.01)	0.90 (0.01)	0.01	0.369
Wide Range Achievement Test‐3–Reading	47.40 (0.12)	48.37 (0.11)	–0.96	3.18E‐09^*^
Hooper Visual Organization Test	24.19 (0.08)	24.48 (0.08)	–0.29	0.010
**NP tests with male advantage**				
Finger Tapping				
Right hand	48.11 (0.22)	42.17 (0.20)	5.94	< 1.0E‐10^*^
Left hand	43.89 (0.19)	38.64 (0.17)	5.25	< 1.0E‐10^*^
Boston Naming Test ^***^				
10 items	9.58 (0.02)	9.36 (0.02)	0.22	< 1.0E‐10^*^
30 items	27.14 (0.08)	26.48 (0.07)	0.67	2.36E‐10^*^
Similarities	15.49 (0.09)	15.31 (0.08)	0.18	0.144
Digit Span				
Forward	6.39 (0.05)	6.22 (0.05)	0.17	0.020
Backward	4.45 (0.05)	4.53 (0.04)	–0.08	0.219
Visual Reproduction				
Immediate Recall	7.88 (0.07)	7.65 (0.06)	0.23	0.016
Delayed Recall	6.90 (0.07)	6.70 (0.07)	0.19	0.050
Recognition	2.70 (0.02)	2.67 (0.02)	0.03	0.392

Abbreviations: BNT, Boston Naming Test; FHS, Framingham Heart Study; NP, neuropsychological; SD, standard deviation; SE, standard error.

*Note*. For NP tests values are age‐ and education‐adjusted means (SEs in parentheses).

^+^ Significance for the association of sex with NP test total scores in the multivariate regression analyses.

* Statistically significant results with Bonferroni correction (*P* < 0.00227).

** FHS Gen 1 (Original) and Gen 2 (Offspring) are largely considered non‐Hispanic White.^31^ ^.^

*** Since October 2018, FHS replaced noose with asparagus as one of the BNT items.

### 3.2 Sex differences in NP total scores and composite process scores

The adjusted means and standard deviation of NP tests for both men and women are presented in Table 1. Among these cognitive tests, women performed significantly better than men in LM (immediate and delayed recall, as well as recognition) (*P* < 1.00E‐10), PAL immediate and delayed recall (*P* < 1.00E‐10), and WRAT‐3 Reading (*P* = 3.18E‐09). On the other hand, men performed significantly better than women in the Finger Tapping test (*P* < 1.00E‐10), and BNT (*P* < 1.00E‐9). Similar trends are observed when both cohorts were analyzed separately (Table S3 and S4 in supporting information).

The analysis of composite process scores for three cognitive functions, stratified by sex, is shown in Table 2. Men tended to have lower composite process scores than women in self‐monitoring and related intrusions/confabulations (*P* < 0.017). In contrast, women had lower composite process scores in abstract thinking (*P* = 0.00427).

**TABLE 2 alz13500-tbl-0002:** Composite process scores, stratified by sex.

Composite process scores	Men (*n* = 1125)	Women (*n* = 1373)	Sex effects^+^	*P* value
Self‐monitoring	0.28 (0.48)	0.36 (0.52)	0.08 (0.02)	0.000094^*^
Abstract thinking	4.51 (1.58)	4.30 (1.59)	–0.17 (0.06)	0.00427^*^
Related intrusions confabulations	0.31 (1.78)	0.53 (1.80)	0.24 (0.07)	0.00144^*^

*Note*. Presented as mean and standard deviation.

^+^ Sex effects are presented as beta estimates and standard deviation derived from linear regression models adjusting for age and education, with men as reference group.

* Statistically significant results with Bonferroni correction (*P* < 0.017).

### 3.3 Sex‐specific association between NP total scores and incident AD, and incident MCI

The mean follow‐up time for men (*n* = 913) was 9.5 years and that of women (*n* = 1092) was 9.9 years. Among these aged ≥ 60 participants (*n* = 2005), 54 men and 92 women developed incident AD dementia during the follow‐up.

In men, LM performance was the only significant predictor for an incident AD dementia hazard model among the NP tests considered (Table 3). Lower LM Immediate Recall total score was associated with higher hazards of AD development in men (hazard ratio [HR] = 0.85, 95% confidence interval [CI]: 0.79–0.91; *P* = 8.96E‐06) and women (HR = 0.88, 95% CI: 0.83–0.93; *P* = 8.80E‐06). Likewise, the respective hazards for LM Delayed Recall total scores were 0.83 (95% CI: 0.78–0.90, *P* = 1.18E‐06) in men and 0.87 (95% CI: 0.82–0.91, *P* = 8.09E‐08) in women. In comparison, lower PAL Immediate Recall total scores were significantly associated with increased future risk of AD in women (HR = 0.84, 95%, 0.78–0.90; *P* = 3.11E‐07) but not in men (HR = 0.92, 95%, 0.84–1.01; *P* = 0.072). A similar trend was observed in women for PAS Delayed Recall (*P* = 1.95E‐04). The total scores for PAL Immediate Recall, PAL Delayed Recall, and BNT were not significantly associated with AD incidence in men. The total scores for WRAT‐3 Reading and Finger Tapping test were not significantly associated with AD incidence in either men or women. Sex‐specific association between the other NP total scores and incident AD can be found in Table S5 in supporting information.

**TABLE 3 alz13500-tbl-0003:** Hazard of AD dementia using NP total scores as predictor, stratified by sex.

NP tests	Men (*n* = 913)			Women (*n* = 1092)		
	HR^+^	95% CI HR	*P* value	HR^+^	95% CI HR	*P* value
Logical memory						
Immediate recall	0.85	0.79, 0.91	8.96E‐06^*^	0.88	0.83, 0.93	8.80E‐06^*^
Delayed recall	0.83	0.78, 0.90	1.18E‐06^*^	0.87	0.82, 0.91	8.09E‐08^*^
Recognition	0.68	0.58, 0.80	1.38E‐06^*^	0.81	0.71, 0.93	0.002^*^
Paired Associate Learning						
Immediate recall	0.92	0.84, 1.01	0.072	0.84	0.78, 0.90	3.11E‐07^*^
Delayed recall	0.79	0.67, 0.95	0.010	0.78	0.69, 0.89	1.95E‐04^*^
Trail A	2.15	1.26, 3.68	0.005	2.26	1.56, 3.26	1.46E‐05^*^
Boston Naming Test						
10 items	0.72	0.48, 1.07	0.106	0.67	0.54, 0.83	2.15E‐04^*^
30 items	0.92	0.84, 1.00	0.048	0.89	0.84, 0.94	5.79E‐05^*^
Wide Range Achievement Test‐3–Reading	1.01	0.95, 1.07	0.789	1.00	0.96, 1.05	0.843
Finger Tapping						
Right hand	0.99	0.94, 1.04	0.657	0.97	0.93, 1.02	0.245
Left hand	0.97	0.92, 1.03	0.375	0.99	0.93, 1.04	0.656

Abbreviations: AD, Alzheimer's disease; CI, confidence interval; HR, hazard ratio; NP, neuropsychological.

*Note*. Fifty‐four men and 92 women developed incident AD dementia during the follow‐up from 2005–2019.

^+^ Cox proportional hazards ratios adjusted for age and education.

* Statistically significant results with Bonferroni correction (*P* < 0.0045).

We further tested the sex‐specific associations between these NP total scores and incident MCI as the outcome (Table S6 in supporting information). While similar trends were observed, none of them attained the adjusted significance level of 0.0045 for the prediction of incident MCI.

### 3.4 Sex‐specific association between NP composite process scores and incident AD, and incident MCI

The loadings of each process measure derived by CFA can be found in Table S1. Table 4 presents the association between composite process scores and incident AD. In men, the composite process scores for self‐monitoring and related intrusions/confabulations were significantly associated with incident AD: every one standard deviation increase in composite process score of self‐monitoring was associated with increased hazards of 1.29 (95% CI: 1.08–1.56, *P* = 0.0063) for AD. A similar trend can be observed in women, for whom the HR is 1.23 (95% CI: 1.05–1.45, *P* = 0.0122). On the other hand, the composite process score of related intrusions/confabulations in men is associated with reduced hazards of incident AD, with an HR of 0.62 (95% CI: 0.47–0.81, *P* = 0.00057) while for women, that of abstract thinking was associated with reduced hazards of incident AD (HR = 0.78, 95% CI: 0.64–0.97) but did not reach the adjusted significance level (*P* = 0.0231).

**TABLE 4:** Hazard of AD dementia using composite process scores as predictor, stratified by sex.

Composite process scores	Men (*n* = 913)			Women (*n* = 1092)		
	HR^+^	95% CI HR	*P* value	HR^+^	95% CI HR	*P* value
Self‐monitoring	1.29	1.08, 1.56	0.0063^*^	1.23	1.05, 1.45	0.0122^*^
Abstract thinking	0.83	0.63 1.09	0.184	0.78	0.64, 0.97	0.0231
Related Intrusions confabulations	0.62	0.47, 0.81	0.00057^*^	0.80	0.65, 0.99	0.0393

Abbreviations: AD, Alzheimer's disease; CI, confidence interval; HR, hazard ratio; NP, neuropsychological.

*Note*. Fifty‐four men and 92 women developed incident AD dementia during the follow‐up from 2005–2019.

^+^ Cox proportional hazards ratios adjusted for age and education.

* Statistically significant results with Bonferroni correction (*P* < 0.0167).

Like the NP total scores, we explored the sex‐specific associations between NP composite process scores and incident MCI as the outcome (Table S7 in supporting information). We found that composite process score for self‐monitoring in females remained significantly associated, after adjusting for multiple testing, with incident MCI, with an HR of 1.51 (95% CI = 1.08–2.10, *P* = 0.015).

### 3.5 Sex‐specific optimal NP profiles for predicting incident AD

**FIGURE 2:**
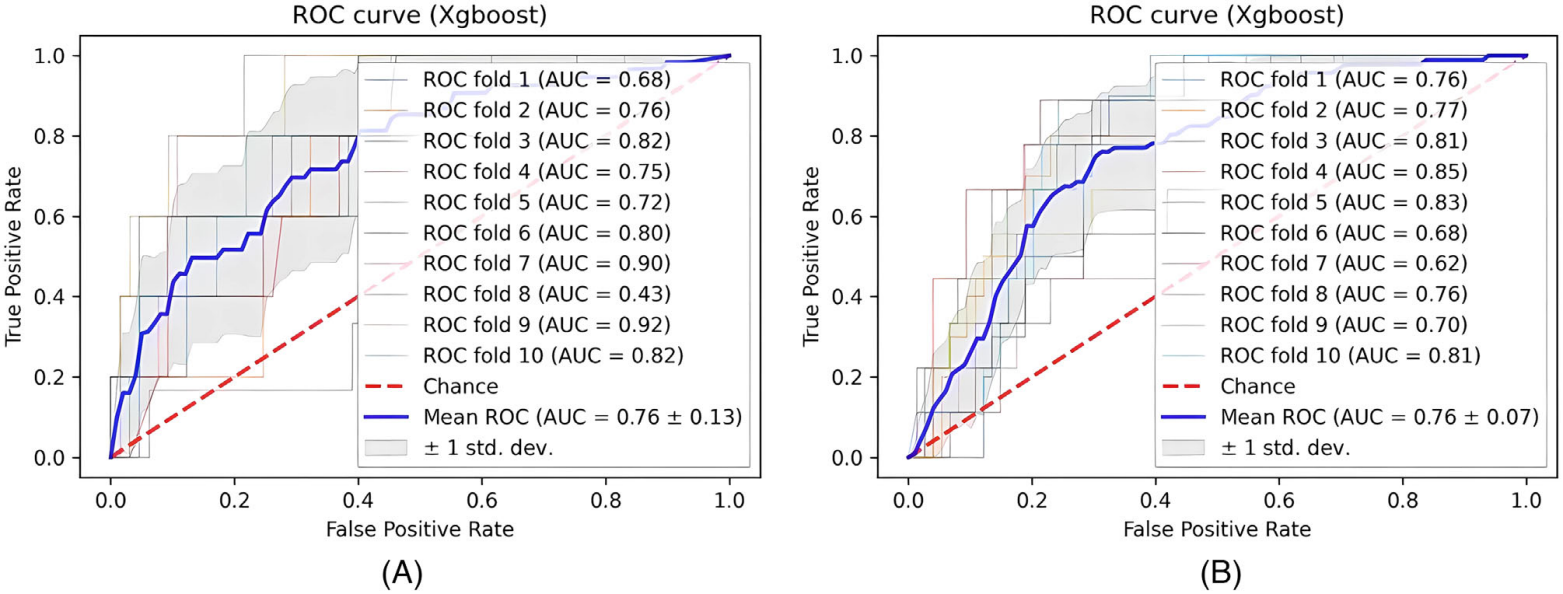
ROC of optimal NP profiles for incident AD prediction in men (A) and women (B). The mean ROC of 10‐folder cross‐validation is shown by the blue line. AD, Alzheimer's disease; AUC, area under the curve; NP, neuropsychological; ROC, receiver operating characteristic

The demographics are shown in Table S8 in supporting information. Figure 2 presents the receiver operating characteristic (ROC) curves of the best models trained on demographic covariates (age and education category), NP summary scores, and composite process scores for men and women. For men, the optimal NP profile included 10 metrics, achieving the best AUC (0.76 ± 0.13) of incident AD prediction (Table 5). For women, the optimal NP profile achieved a comparable AUC (0.76 ± 0.07) of incident AD prediction with eight metrics. Two composite process scores—self‐monitoring and related intrusions/confabulations—are included in the optimal NP profile for men but not for women. Age was ranked as the third important metric for women aged ≥ 65 years.

**TABLE 5:** The optimal NP profiles for incident AD prediction in men and women.

Importance order	Men	Women
1	Age	Trails A
2	Paired Associate Learning Recognition	Paired Associate Learning Recognition
3	Composite process score for self‐monitoring	Age
4	Trails A	Boston Naming Test 10 items
5	Logical memory delayed recall	Visual reproduction delayed recall
6	Verbal fluency test category	Trails B
7	Visual reproduction recognition	Verbal fluency test category
8	Logical memory recognition	Verbal fluency test phonemic
9	Paired Associate Learning Immediate Recall	–
10	Composite process score for related intrusions confabulations	–

Abbreviations: AD, Alzheimer's disease; NP, neuropsychological.

## 4 DISCUSSION

Sex differences in NP test performance have important implications for early diagnosis of AD. Using a robust NP dataset from FHS, this study explored the effects of sex‐specific cognitive performance for both conventional total scores and novel composite process scores—derived from BPA process measures—in three distinct approaches: (1) cross‐sectional comparison (2) time‐to‐event analysis, and (3) predictive modeling.

This study reaffirmed the findings of previous studies, including the increasing level of education^49^ and overall better NP performance across generations (reminiscent of the Flynn effect;^50^ see Tables S3 and S4). In addition, consistent sex differences were found in the total scores of LM and PAL (with women outperforming men) and BNT and Finger Tapping (with men outperforming women).^6^, ^7^, ^51^ Particularly, sex differences in LM and PAL performance in individuals who were dementia free were consistently significant across three generations, as previously reported in other studies.^22^

The results from the Cox proportional hazards models suggested that sex‐specific NP performance in selected tests may be important in incident AD modeling and potentially incident MCI as well. Specifically, impairments in language function—a key assessment of BNT—is a more sensitive tool to detect early cognitive perturbations in women than men. As this is an NP test with male advantage, one could speculate that this is not ideal for early detection of preclinical AD in men, perhaps due to ceiling effects.

Similarly, we found that the use of total scores in PAL could identify preclinical female AD cases better. Even though PAL is an NP test advantaged toward women, our data do not support the idea of a ceiling effect in the ability of this test to predict AD. The underlying reasons for such observation remain unclear. It is possible that a subset of preclinical AD women continued to perform within current norms by “masking” their cognitive deficits using compensatory strategies and hence, it should be interpreted as women with better PAL performances are less at risk for incident AD.

To test the hypothesis of compensatory mechanism, this study included an analysis of sex differences in process measures as well. Confirmatory factor analysis was used to generate composite process scores for three categories of cognitive phenotypes, to provide a better understanding of the underlying cognitive architecture in these process measures. The results from cross‐sectional comparisons suggest that men and women used different strategies to tackle the NP tests. For example, while women have significantly higher composite process scores for self‐monitoring and related intrusions/confabulations, men have that for abstract thinking. In both men and women, the composite process score for self‐monitoring was significantly associated with increased hazards of incident AD. Interestingly, an increase in related intrusions/confabulations seems to confer protection against incident AD. This trend was observed for both men and women but was only significant in men (*P* = 0.00057).

Similar associations of lesser effect sizes were observed for the outcome of incident MCI, which can be attributed to the limited follow‐up time, as the accrued person‐years for incident MCI was shorter compared to incident AD due to censoring. It has also been suggested that MCI is harder to diagnose in women with current NP scales.^23^ We suggest future research to collect NP test performance data, both correct and process responses, from a younger population to better understand the implications of these sex differences.

The results demonstrate that sex differences exist in both NP total scores and process measures, and they can be leveraged for better prediction of incident AD in men and women. Our machine learning models identified the optimal NP profiles for incident AD prediction in men and women and ranked them in accordance with importance. While age remained the most important metric for men aged ≥ 65 years, it was ranked third, behind Trails A and PAL–Recognition, for the female counterparts. BNT is not part of the optimal NP profile for men; it is, however, the fourth important measure for women's optimal NP profile. Similarly, the combination of conventional total scores and novel process scores seems to yield more information than the former alone in men, but not in women (e.g., composite process scores for self‐monitoring and related intrusions/confabulations were included in the optimal NP profiles for men only). These results from the predictive modeling are consistent with those of the Cox proportional hazards model, and more importantly, demonstrated the need to look beyond the face value of sex differences in conventional NP scores. While a few studies hypothesized that rapid disease progression in AD women may be attributed to their abilities to “mask” their early cognitive impairment signs with compensatory mechanisms during the preclinical AD phase,^22^ the results of this study suggested that these compensatory mechanisms, as represented by the composite process scores, may play a more important role in AD detection in men, rather than women. This finding re‐emphasized the need for researchers to reassess the NP testing process, evaluating the process measures on top of the standard approach of scoring only correct responses. These process measures may reveal subtle performance deficits and compensatory strategies that occur in pre‐clinical phases of the AD spectrum, which otherwise could have gone undetected with standard scales currently used in clinical practice. Given the recent US Food and Drug Administration regulation change for AD drugs’ evaluation,^52^ leading to the approval of lecanemab,^53^ it highlights the importance of sensitive cognitive assessment tools to detect cognitive changes during preclinical AD. Given early diagnosis is critical for the successful initiation of therapeutic interventions, the consideration and implementation of sex‐specific NP indices into these assessment metrics may be of clinical relevance.

There are several limitations to this study. The FHS participants are relatively well educated, and Gen 1 and 2 cohorts are primarily non‐Hispanic White, which limits the generalizability of the results for other populations. Cohort differences in cognitive performance are also observed (Tables S3 and S4) and were illustrated in previous studies.^54^, ^55^, ^56^ In addition, unlike the results displayed in Table 1 in which participants were naïve to FHS NP tests, the results in Table 2 may be affected by practice effects as some participants had undergone assessments prior to BPA implementation in 2005. As FHS is an ongoing cohort study, high‐risk cases are flagged through dementia surveillance and prioritized for clinical consensus panel diagnosis, instead of biomarker‐based diagnosis as in other research settings. Therefore, it is possible that some individuals within the non‐demented group could have undiagnosed MCI, which may lead to non‐differential misclassification bias in both sexes—the true effects are likely to be greater in magnitude compared to the reported effects. Finally, while there is no significant statistical interaction observed, it is important to demonstrate the effect modification by sex in various NP tests, in both total scores and process measures. Further research is warranted, including survival modelling with competing risks such as death and more AD risk factors, to design more personalized screening batteries for more heterogeneous populations.

## 5 CONCLUSION

The results of this study suggest that sex differences in NP may be leveraged to develop more sensitive indices for the diagnosis of AD. While these results warrant validation in additional cohorts, future studies will be necessary to (1) identify the biological underpinning of such sex‐related differences in performance and strategy; (2) extend the use of composite process scores in preclinical AD and AD research; (3) establish sex‐specific process patterns between men and women and their correlation with preclinical AD and incident AD; (4) integrate sex differences in total scores and composite process scores in risk prediction models of incident MCI and AD, alongside known risk factors; (5) identify socioeconomic factors that might affect sex differences in NP performance across geographical areas and generations; and (6) follow the trajectory of total and process scores longitudinally. It should be noted that technological advances will allow process characterization that will not only automate the process scores reported in this study, but also extend the sensitivity of process scores exponentially by generating metrics that manual scoring cannot produce.

## CONFLICT OF INTEREST STATEMENT

M.T.F. is the co‐founder of the Women's Brain Project. In the past 2 years she has received consulting and speaking fees from Roche, Eli Lilly, and Lundbeck unrelated to this project. A.S.C. is an official employee of Altoida and works as Chief Medical Officer. She is also co‐founder and pro bono CEO of the Women's Brain Project. R.A. is a scientific advisor to Signant Health and a scientific consultant to Biogen and the Davos Alzheimer's Collaborative (DAC). She also serves as Director of the Global Cohort Program for DAC. The other authors declare no conflicts of interest. Author disclosures are available in the supporting information.

## CONSENT STATEMENT

All participants provided written informed consent.

## Supporting information

Supporting Information

Supporting Information
