# Supplementary material for: Maximizing utility of neuropsychological measures in sex‐specific predictive models of incident Alzheimer's disease in the Framingham Heart Study
Source: Alzheimers Dement. 2023 Oct 26;20(2):1112–22. doi: 10.1002/alz.13500 (PMC10917035; doi:10.1002/alz.13500)
Supplement: Supplementary file 1 — Supporting Information [file ALZ-20-1112-s002.docx]

**Table A1: The Description of Error Measures Used in This Study**

| **Cognitive function** | **Error measures** | **NP test** | **Test description** | **Error description** | **Derived loadings** | **Missing rate** |
| --- | --- | --- | --- | --- | --- | --- |
| Self-Monitoring | CmC_SC | Clock Drawing Test | The participant is asked to **draw a clock** | If they make an error and they make an attempt to correct it, regardless of whether the final production is correct overall. | 0.410 | 3.1% |
|  | CpC_SC | Clock Drawing Test | The participant is asked to **copy a clock** | If they make an error and they make an attempt to correct it, regardless of whether the final production is correct overall. | 0.632 | 4.1% |
|  | TA_SC_Perceptual | Trail Making Test | Participant is asked to complete a task that requires them to draw a line connecting circles in numerical order (similar to “connect the dots”) | A perceptual error occurs when they go to the wrong circle and the number looks similar to the correct number (e.g., “1” and “7”); this variable captures the situation where the participant makes that type of error but spontaneously corrects it. | 0.014 | 4.9% |
|  | TA_SC_Other |  |  | This variable captures the situation where the participant makes an error other than a perceptual error (which is described for TA_SC_P), but spontaneously corrects it | 0.574 | 4.9% |
|  | TB_SC_Perceptual | Trail Making Test | Participant is asked to complete a task that requires them to draw a line connecting circles by alternating between numbers and letters (i.e., 1-A-2-B) | A perceptual error occurs when they go to the wrong circle and the number/letter looks similar to the correct letter/number (e.g., “B” and “8”); this variable captures the situation where the participant makes that type of error but spontaneously corrects it. | 0.502 | 12.0% |
|  | TB_SC_Other |  |  | A loss-of-set error occurs when they fail to switch over to the other sequence (e.g., 1-A-2-B-C); a sequencing error occurs when the participant correctly switches to either a number or letter, but the circle they go to is not the correct number/letter in the sequence (e.g., 1-A-2-C); this variable captures the total number of loss-of-set and sequencing errors that are spontaneously corrected by the participant. | 0.636 | 12.0% |
| Abstract Thinking | Sim_CC | Similarities | Participant is given two words and has to say in what way the two words are similar (there are 13 sets of words). | The response for a set of words can be scored as “2” (the most abstract), “1” (concrete), or “0” (incorrect). This variable captures the number of items (out of 13) for which the response earned a credit of “**2**.” | 0.530 | 1.4% |
|  | Sim_PC |  |  | The response for a set of words can be scored as “2” (the most abstract), “1” (concrete), or “0” (incorrect). This variable captures the number of items (out of 13) for which the response was concrete, but accurate, and earns a score of “**1**.” | 0.885 | 1.4% |
|  | Sim_0pt_C |  |  | The response for a set of words can be scored as “2” (the most abstract), “1” (concrete), or “0” (incorrect). This variable captures the number of items (out of 13) for which the response was earned a score of “**0**” and the error is considered “concrete.” | 0.444 | 1.4% |
| Related Intrusions Confabulations | LM_I_RC | Logical Memory | Participant is read a story and asked to repeat it; | This is the total number of confabulations (new details added or mixing up details thus changing the meaning) that are conceptually related to the original story details. | 0.955 | 0.9% |
|  | LM_I_RI |  |  | This is the total number of intrusions (new details added that are associated with something within the testing session) that are conceptually related to the original story details | 1.056 | 0.9% |
|  | LM_D_NRC | Logical Memory | After a delay (filled with other activities), the participant is asked to recall the story that was previously read to them | This is the total number of conceptually related confabulations (see LM_I_RC) that are produced during this delayed condition that were NOT produced during the immediate recall. | -0.851 | 1.6% |
|  | LM_D_NRI |  |  | This is the total number of conceptually related intrusions (see LM_I_RI) that are produced during this delayed condition that were NOT produced during the immediate recall. | -0.475 | 1.6% |
|  | VR_I_Contamination | Visual Reproductions | Participants are shown a total of four designs; the design(s) is then hidden and the participant is asked to draw the design from memory | Contamination from another design means that features from one of the other four designs is incorrectly incorporated into the participant’s drawing. | -0.739 | 2.7% |
|  | VR_D_Contamination | Visual Reproductions | After a delay (filled with other activities), the participant again asked to produce the four drawings that were previously presented | Contamination from another design means that features from one of the other four designs is incorrectly incorporated into the participant’s drawing. | 0.918 | 3.4% |
|  | PA_T1_Int | Paired Associates | Participant is given three trials to learn a set of 10 word pairs that are presented orally; during recall the participant is given the first word of each pair (“stimulus word”) and asked to say the associated word | PAL interference errors are when the participant gives a response that is one of the words previously presented to them, but is NOT the word that was paired with the stimulus word; this variable refers to the number of these errors during the **first** learning trial. | 0.896 | 7.8% |
|  | PA_T2_Int |  |  | PAL interference errors are when the participant gives a response that is one of the words previously presented to them, but is NOT the word that was paired with the stimulus word; this variable refers to the number of these errors during the **second** learning trial. | -0.613 | 7.8% |
|  | PA_T3_Int |  |  | PAL interference errors are when the participant gives a response that is one of the words previously presented to them, but is NOT the word that was paired with the stimulus word; this variable refers to the number of these errors during the **third** learning trial. | -0.069 | 7.8% |
|  | PA_T1_Rel |  |  | “Related” errors occur when the participant gives a word that is NOT one of the words previously presented, but the word they give is conceptually related to the stimulus word; this variable refers to the number these errors during the **first** learning trial. | -0.491 | 7.8% |
|  | PA_T2_Rel |  |  | “Related” errors occur when the participant gives a word that is NOT one of the words previously presented, but the word they give is conceptually related to the stimulus word; this variable refers to the number these errors during the **second** learning trial. | -0.538 | 7.8% |
|  | PA_T3_Rel |  |  | “Related” errors occur when the participant gives a word that is NOT one of the words previously presented, but the word they give is conceptually related to the stimulus word; this variable refers to the number these errors during the **third** learning trial. | -0.132 | 7.8% |
|  | PA_D_Int | Paired Associates | After a delay (filled with other activities), the participant is again given each of the 10 stimulus words (first word of the pair) from the previously presented pairs of words and asked to say the associated word | PAL interference errors are when the participant gives a response that is one of the words previously presented to them, but is NOT the word that was paired with the stimulus word; this variable refers to the number of these errors during the delayed recall trial. | 0.263 | 6.6% |
|  | PA_D_Rel |  |  | “Related” errors occur when the participant gives a word that is NOT one of the words previously presented, but the word they give is conceptually related to the stimulus word; this variable refers to the number these errors during the delayed recall trial. | -0.097 | 6.6% |

**Table A2: Non-demographic Characteristics at Baseline of Participants Without Dementia**

| **Variables** | **Gen 1 & 2 participants without dementia (n=4015)** | | |
| --- | --- | --- | --- |
|  | **Men (n=1787)** | **Women (n=2228)** | ***P* values** |
| Diabetes, n (%) | 234 (13.1) | 154 (6.9) | <.0001 |
| Hypertension, n (%) | 694 (38.8%) | 797 (35.8) | 0.055 |
| Smoking status, n (%) | 161 (9.0) | 193 (8.7) | 0.774 |

**Table A3: Demographic, and Adjusted Means of Neuropsychological Test Total Scores at Baseline of Gen 1 (Original) Participants Without Dementia**

| Variables | Men (n=404) | Women (n=622) | **Effect size** | ***P* values^+^** |
| --- | --- | --- | --- | --- |
| Age, years, mean ± SD  (Range) | 77.6±6.9 | 80.5±7.1 | - | <.0001 |
| Education, n (%) |  |  |  | 0.925 |
| High school graduate and below | 278(68.8) | 425(68.3) | - |  |
| Beyond high school graduate | 126(31.2) | 197(31.7) | - |  |
| Race/ethnicity^**^, n (%) |  |  | - | 1.0 |
| Non-Hispanic white | 404 (100) | 622 (100) |  |  |
| Others | 0 | 0 |  |  |
| **NP tests with female advantage** |  |  |  |  |
| Logical Memory |  |  |  |  |
| Immediate Recall | 8.25(0.19) | 9.44(0.15) | -1.18 | 6.90E-07^*^ |
| Delayed Recall | 6.99(0.20) | 7.97(0.16) | -0.98 | 9.02E-05^*^ |
| Recognition | 8.78(0.17) | 9.08(0.12) | -0.31 | 0.139 |
|  |  |  |  |  |
| Paired Associate Learning |  |  |  |  |
| Immediate Recall | 11.43(0.17) | 12.79(0.14) | -1.36 | 3.61E-10^*^ |
| Delayed Recall | 6.75(0.16) | 7.59(0.11) | -0.84 | 3.17E-05^*^ |
| Recognition | 8.88(0.72) | 9.04(0.43) | -0.16 | 0.855 |
|  |  |  |  |  |
| Verbal Fluency Test |  |  |  |  |
| Phonemic (FAS) | 29.70(0.67) | 31.43(0.55) | -1.73 | 0.041 |
| Category (Animals) | 12.93(1.91) | 12.08(1.11) | 0.85 | 0.728 |
|  |  |  |  |  |
| Trails – Log-transformed |  |  |  |  |
| Trail A | 0.65(0.02) | 0.61(0.02) | 0.04 | 0.171 |
| Trail B | 1.30(0.05) | 1.32(0.03) | -0.02 | 0.704 |
|  |  |  |  |  |
| Wide Range Achievement Test-3 – Reading | 47.00(0.57) | 48.47(0.40) | -1.47 | 0.035 |
|  |  |  |  |  |
| Hooper Visual Organization Test | 19.39(0.51) | 19.52(0.36) | -0.14 | 0.824 |
|  |  |  |  |  |
| **NP tests with male advantage** |  |  |  |  |
| Finger Tapping |  |  |  |  |
| Right Hand | 38.62(1.08) | 34.33(0.75) | 4.29 | 0.001^*^ |
| Left Hand | 35.85(0.97) | 32.13(0.66) | 3.72 | 0.002^*^ |
|  |  |  |  |  |
| Boston Naming Test^***^ |  |  |  |  |
| 10 items | 9.24(0.07) | 8.88(0.06) | 0.36 | 5.42E-05^*^ |
| 30 items | 24.41(0.59) | 23.36(0.41) | 1.04 | 0.145 |
|  |  |  |  |  |
| Similarities | 13.38(0.27) | 13.26(0.22) | 0.12 | 0.724 |
| Digits Span |  |  |  |  |
| Forward | 6.29(0.07) | 6.04(0.06) | 0.25 | 0.004 |
| Backward | 4.26(0.06) | 4.41(0.05) | -0.15 | 0.062 |
|  |  |  |  |  |
| Visual Reproduction |  |  |  |  |
| Immediate Recall | 5.40(0.16) | 5.30(0.13) | 0.09 | 0.655 |
| Delayed Recall | 4.29(0.16) | 4.07(0.13) | 0.22 | 0.260 |
| Recognition | 1.98(0.06) | 1.95(0.05) | 0.03 | 0.715 |

For NP tests values are age- and education-adjusted means (SEs in parentheses).

^+^ Significance for the association of sex with Neuropsychological Test total scores in the multivariate regression analyses.

^*^ Statistically significant results with Bonferroni correction (p < 0.00227).

^**^ FHS Gen 1 (Original) is largely considered as non-Hispanic white.

^***^ Since October 2018, FHS replaced noose with asparagus as one of the BNT items.

**Table A4: Demographic, and Adjusted Means of Neuropsychological Test Total Scores at Baseline of Gen 2 (Offspring) Participants Without Dementia**

| Variables | Men (n=1383) | Women (n=1606) | **Effect size** | ***P* values^+^** |
| --- | --- | --- | --- | --- |
| Age, years, mean ± SD  (Range) | 62.5±9.6 | 62.4±9.9 | - | 0.747 |
| Education, n (%) |  |  |  | 0.001 |
| High school graduate and below | 445(32.2) | 609(37.9) | - |  |
| Beyond high school graduate | 938(67.8) | 997(62.1) | - |  |
| Race/ethnicity^**^, n (%) |  |  | - | 0.932 |
| Non-Hispanic white | 1361 (98.4) | 1580 (98.4) |  |  |
| Others | 22 (1.6) | 26 (1.6) |  |  |
| **NP tests with female advantage** |  |  |  |  |
| Logical Memory |  |  |  |  |
| Immediate Recall | 10.60(0.09) | 11.51(0.08) | -0.91 | < 1.0E-10^*^ |
| Delayed Recall | 9.61(0.10) | 10.56(0.09) | -0.95 | < 1.0E-10^*^ |
| Recognition | 9.22(0.03) | 9.52(0.03) | -0.30 | < 1.0E-10^*^ |
|  |  |  |  |  |
| Paired Associate Learning |  |  |  |  |
| Immediate Recall | 12.74(0.09) | 14.22(0.08) | -1.48 | < 1.0E-10^*^ |
| Delayed Recall | 7.86(0.04) | 8.43(0.04) | -0.57 | < 1.0E-10^*^ |
| Recognition | 9.68(0.06) | 9.73(0.05) | -0.06 | 0.470 |
|  |  |  |  |  |
| Verbal Fluency Test |  |  |  |  |
| Phonemic (FAS) | 34.68(0.83) | 36.85(0.79) | -2.17 | 0.055 |
| Category (Animals) | 18.18(0.42) | 17.76(0.39) | 0.42 | 0.449 |
|  |  |  |  |  |
| Trails – Log-transformed |  |  |  |  |
| Trail A | 0.45(0.00) | 0.43(0.00) | 0.02 | 0.001^*^ |
| Trail B | 0.89(0.01) | 0.87(0.01) | 0.02 | 0.123 |
|  |  |  |  |  |
| Wide Range Achievement Test-3 – Reading | 47.41(0.13) | 48.31(0.12) | -0.90 | 6.73E-08^*^ |
|  |  |  |  |  |
| Hooper Visual Organization Test | 24.53(0.08) | 24.90(0.08) | -0.37 | 0.001^*^ |
|  |  |  |  |  |
| **NP tests with male advantage** |  |  |  |  |
| Finger Tapping |  |  |  |  |
| Right Hand | 48.79(0.23) | 42.80(0.21) | 5.99 | < 1.0E-10^*^ |
| Left Hand | 44.45(0.20) | 39.12(0.18) | 5.33 | < 1.0E-10^*^ |
|  |  |  |  |  |
| Boston Naming Test^***^ |  |  |  |  |
| 10 items | 9.71(0.02) | 9.56(0.02) | 0.16 | 1.44E-08^*^ |
| 30 items | 27.35(0.07) | 26.77(0.07) | 0.58 | 2.51E-09^*^ |
|  |  |  |  |  |
| Similarities | 16.33(0.09) | 16.11(0.08) | 0.21 | 0.083 |
| Digits Span |  |  |  |  |
| Forward | 6.59(0.09) | 6.52(0.08) | 0.08 | 0.517 |
| Backward | 4.80(0.08) | 4.73(0.08) | 0.07 | 0.510 |
|  |  |  |  |  |
| Visual Reproduction |  |  |  |  |
| Immediate Recall | 8.71(0.08) | 8.43(0.07) | 0.28 | 0.008 |
| Delayed Recall | 7.75(0.09) | 7.56(0.08) | 0.20 | 0.086 |
| Recognition | 2.94(0.03) | 2.92(0.02) | 0.02 | 0.492 |

For NP tests values are age- and education-adjusted means (SEs in parentheses).

^+^ Significance for the association of sex with Neuropsychological Test total scores in the multivariate regression analyses.

^*^ Statistically significant results with Bonferroni correction (p < 0.00227).

^**^ FHS Gen 2 (Offspring) is largely considered as non-Hispanic white.

^***^ Since October 2018, FHS replaced noose with asparagus as one of the BNT items.

**Table A5: Hazard of AD Dementia using Total Scores of Non-Significant Sex Difference NP as Predictor,**

**Stratified by Sex**

| **NP tests** | **Men (n=913)** | | | **Women (n=1092)** | | |
| --- | --- | --- | --- | --- | --- | --- |
|  | **HR^+^** | **95% CI HR** | ***P* value** | **HR^+^** | **95% CI HR** | ***P* value** |
| Verbal Fluency Test |  |  |  |  |  |  |
| Phonemic (FAS) | 0.99 | 0.97, 1.01 | 0.378 | 0.98 | 0.96, 1.00 | 0.015 |
| Category (Animals) | 0.92 | 0.87, 0.98 | 0.013 | 0.89 | 0.84, 0.94 | 9.75E-06^*^ |
| Trail B | 1.19 | 1.07, 1.32 | 0.002^*^ | 1.18 | 1.07, 1.30 | 7.83E-04^*^ |
| Similarities | 0.90 | 0.84, 0.96 | 0.002^*^ | 0.95 | 0.90, 1.00 | 0.038 |
| Paired Associate Learning Recognition | 0.74 | 0.62, 0.88 | 6.48E-04^*^ | 0.73 | 0.62, 0.84 | 3.47E-05^*^ |
| Digits Span |  |  |  |  |  |  |
| Forward | 1.07 | 0.87, 1.33 | 0.522 | 0.95 | 0.81, 1.13 | 0.575 |
| Backward | 0.95 | 0.76, 1.17 | 0.613 | 0.94 | 0.79, 1.13 | 0.589 |
| Hooper Visual Organization Test | 0.94 | 0.88, 1.01 | 0.080 | 0.90 | 0.85, 0.95 | 5.28E-05^*^ |
| Visual Reproduction |  |  |  |  |  |  |
| Immediate Recall | 0.86 | 0.78, 0.96 | 0.005 | 0.83 | 0.76, 0.90 | 1.07E-05^*^ |
| Delayed Recall | 0.82 | 0.74, 0.91 | 2.21E-04^*^ | 0.81 | 0.74, 0.87 | 2.33E-07^*^ |
| Recognition | 0.65 | 0.51, 0.82 | 3.04E-04^*^ | 0.71 | 0.59, 0.86 | 3.40E-04^*^ |

^+^ Cox proportional hazards ratios adjusted for age and education.

^*^ Statistically significant results with Bonferroni correction (p < 0.0045).

**Table A6: Hazard of MCI Incidence using NP Total Scores as Predictor, Stratified by Sex**

| **NP tests** | **Men (n=826)** | | | **Women (n=983)** | | |
| --- | --- | --- | --- | --- | --- | --- |
|  | **HR^+^** | **95% CI HR** | ***P* value** | **HR^+^** | **95% CI HR** | ***P* value** |
| Logical Memory |  |  |  |  |  |  |
| Immediate Recall | 0.98 | 0.92, 1.04 | 0.473 | 0.98 | 0.93, 1.03 | 0.350 |
| Delayed Recall | 0.97 | 0.91, 1.03 | 0.281 | 0.98 | 0.94, 1.03 | 0.438 |
| Recognition | 0.92 | 0.77, 1.08 | 0.298 | 1.05 | 0.90, 1.22 | 0.536 |
| Paired Associate Learning |  |  |  |  |  |  |
| Immediate Recall | 0.94 | 0.87, 1.01 | 0.084 | 0.95 | 0.90, 1.01 | 0.087 |
| Delayed Recall | 0.90 | 0.77, 1.04 | 0.162 | 0.92 | 0.81, 1.05 | 0.226 |
| Trail A | 2.40 | 1.30, 4.42 | 0.0052 | 1.60 | 0.91, 2.82 | 0.104 |
| Boston Naming Test |  |  |  |  |  |  |
| 10 items | 0.86 | 0.56, 1.02 | 0.479 | 0.81 | 0.64, 1.02 | 0.073 |
| 30 items | 0.94 | 0.87, 1.06 | 0.138 | 0.96 | 0.90, 1.02 | 0.170 |
| Wide Range Achievement Test-3 – Reading | 0.98 | 0.93, 1.04 | 0.318 | 0.99 | 0.95, 1.04 | 0.803 |
| Finger Tapping |  |  |  |  |  |  |
| Right Hand | 1.01 | 0.97, 1.31 | 0.486 | 1.02 | 0.98, 1.06 | 0.339 |
| Left Hand | 1.00 | 0.96, 1.02 | 0.970 | 1.01 | 0.97, 1.06 | 0.595 |

81 men (time to diagnosis: 5.2±2.8 years) and 119 women (time to diagnosis: 5.6±3.1 years) developed incident MCI during the follow-up from 2005 – 2019.

^+^ Cox proportional hazards ratios adjusted for age and education, with no statistically significant results after Bonferroni correction (p < 0.0045).

**Table A7: Hazard of MCI Incidence using Composite Process Scores as predictor, Stratified by Sex**

| **Composite process scores** | **Men(n=826)** | | | **Women(n=983)** | | |
| --- | --- | --- | --- | --- | --- | --- |
|  | **HR^+^** | **95% CI HR** | ***P* value** | **HR^+^** | **95% CI HR** | ***P* value** |
| Self-Monitoring | 1.23 | 0.85, 1.80 | 0.272 | 1.51 | 1.08, 2.10 | 0.015* |
| Abstract Thinking | 0.91 | 0.79, 1.05 | 0.194 | 0.97 | 0.86, 1.10 | 0.632 |
| Related Intrusions Confabulations | 0.93 | 0.82, 1.06 | 0.274 | 0.99 | 0.89, 1.10 | 0.877 |

81 men (time to diagnosis: 5.2±2.8 years) and 119 women (time to diagnosis: 5.6±3.1 years) developed incident MCI during the follow-up from 2005 – 2019.

^+^ Cox proportional hazards ratios adjusted for age and education.

^*^ Statistically significant results with Bonferroni correction (p < 0.0167).

**Table A8: Demographics of Participants for Optimal NP Profile in Predicting AD**

| Variables | Men (n=697) | Women (n=829) | ***P* values** |
| --- | --- | --- | --- |
| Age, years, mean ± SD (Range)^*^ | 74.3±7.0  (65, 100) | 75.3±7.9  (65, 98) | 0.010 |
| Education, n (%) |  |  | 0.009 |
| High school graduate and below | 221 (31.7) | 317 (38.2) |  |
| Beyond high school graduate | 476 (68.3) | 512 (61.8) |  |
| Time to diagnosis | 5.2 (3.1) | 5.6 (3.4) | 0.505 |

51 men (time to diagnosis: 5.2±3.1 years) and 92 women (time to diagnosis: 5.6±3.4 years) developed incident AD dementia during the follow-up from 2005 – 2019.

^*^ Participants are aged 65 and above to minimize class imbalance and optimize the model performance.
